# Evaluation of different bacterial honey isolates as probiotics and their efficient roles in cholesterol reduction

**DOI:** 10.1007/s11274-022-03259-8

**Published:** 2022-05-04

**Authors:** Noura O. Abdelsamad, Mona A. Esawy, Zeinab E. Mahmoud, Asmaa I. El-Shazly, Tarek R. Elsayed, Amira A. Gamal

**Affiliations:** 1 Biotechnology Program, Faculty of Agriculture, Cairo University, Giza, Egypt; 2 Chemistry of Natural and Microbial Products Department, Pharmaceutical and Drug Industries Research Institute, National Research Centre, Dokki, Cairo, 12622 Egypt; 3 Department of Microbiology, Faculty of Agriculture, Cairo University, Giza, Egypt; 4 The American University in Cairo, New Cairo, 11835 Egypt

**Keywords:** Antimicrobial, Cholesterol oxidase, Honey isolates, Levan, Levansucrase, Probiotic activity

## Abstract

Continue to hypothesize that honey is a storehouse of beneficial bacteria, and the majority of these isolates are levansucrase producers. Accordingly, ten bacterial strains were isolated from different honey sources. Four honey isolates that had the highest levansucrase production and levan yield were identified by the partial sequencing of the 16S rRNA gene as *Achromobacter *sp. (10A), *Bacillus paralicheniformis* (2M), *Bacillus subtilis* (9A), and *Bacillus paranthracis* (13M). The cytotoxicity of the selected isolates showed negative blood hemolysis. Also, they are sensitive to the tested antibiotics (Amoxicillin + Flucloxacillin, Ampicillin, Gentamicin, Benzathine benzylpenicillin, Epicephin, Vancomycin, Amikacin, and Zinol). The isolates had strong alkaline stability (pHs 9, 11) and were resistant to severe acidic conditions (29–100 percent). The tested isolates recorded complete tolerance to both H_2_O_2_ and the bile salt (0.3% Oxgall powder) after 24 h incubation. The cell-free supernatant of the examined strains had antifungal activities against *C. Albicans* with varying degrees. Also, isolates 2M and 13M showed strong activities against *S. aureus*. The isolates showed strong adhesion and auto-aggregation capacity. Isolate 10A showed the highest antioxidant activity (91.45%) followed by 2M (47.37%). The isolates recorded different catalase and protease activity. All isolates produced cholesterol oxidase and lipase with different levels. Besides, the four isolates reduced LDL (low-density lipoprotein) to different significant values. The cholesterol-reducing ability varied not only for strains but also for the time of incubation. The previous results recommended these isolates be used safely in solving the LDL problem.

## Introduction

Probiotics are a group of microorganisms that give a health benefit to the host when present in enough amounts (Hill and Sanders 2013). Probiotic bacteria are the balance of human health with many benefits. They can lower cholesterol in the body, improve the immune system, stimulate the digestion process, and nutrient absorbance (Antushevich 2020). However, few studies examined the probiotics to act as an alternative antimicrobial therapy or as a new antibiotics source (Silva et al. 2020). There are criteria that beneficial bacteria should meet, the most important of which is the absence of cytotoxicities like hemolysis and antibiotic resistance. Besides, the bacteria should tolerate the stomach hard environment (severe acidity). Like, low pH and high bile salt concentration. The capacity of the bacteria to adhere to the produced mucus by intestinal epithelium is an important criterion for selecting probiotics. These criteria increase their probability of survival in the gastrointestinal tract and thus permit bacteria to appear their positive health effects (García-Cayuela et al. 2014). Previously, *Achromobacter* sp. was identified as a probiotic bacterium that influences plant functional traits. Also, it was isolated from bees’ gut microbiota (Wang et al. 2015). *Bacillus paralicheniformis* was isolated from the grass carp intestine and was characterized as a probiotic strain (Zhao et al. 2020). Probiotic microbes are characterized by unique behavior in expressing their ability to produce polysaccharides as important health-promoting factors (Angelin and Kavitha 2020). *Bacillus paranthracis* ICIS-279 and *B. subtilis* are probiotics and could be used as models for host-microbiota (gut microbiota) interaction studies (Bukharin et al. 2019; Hamdy et al. 2017). Levan is considered one of the most important polysaccharides playing a significant role in increasing stomach beneficial bacteria. A few years ago, honey has been described as a new source of probiotic bacteria. Also, honey known as a new reservoir for bacteria had potential antimicrobial metabolites (Brudzynski 2021, Lee et al. 2008). Most bees honey isolates are distinguished by the formation of levan-type-fructan (Gamal et al 2021). Accordingly, recent research selected the tested probiotic bacteria based on their levansucrase production and the amount of the levan polymer (Hamdy et al. 2017, 2018; Mostafa et al. 2018). Levan polysaccharides had several applications, such as prebiotic, anti-cancer, and antioxidant agents. An antioxidant can be broadly defined as any substance that delays or inhibits oxidative damage to a target molecule (Yamagishi and Matsui 2011). Till this moment there is scarce information about the antioxidant property of the Bacillus species. One of the most characteristic properties of probiotic bacteria is producing lipase and cholesterol oxidase enzymes. Lipases are a subclass of esterase that catalyzes lipid hydrolysis and plays a significant role in fat food digestion and transport (Jaeger and Eggert 2002). The lipase role is also essential in some medication’s mechanisms like lowering cholesterol, which is the direct cause of clogged heart arteries and represented the main reason in most cardiovascular pathogenesis (Pirahanchi and Sharma 2019). There is a firm relation between high lipid content including cholesterol and the development of cancers such as colon, prostate, breast, etc. (Beloribi-Djefaflia et al. 2016; Pelton et al. 2012; Dos Santos 2014). Therefore, therapies that caused cholesterol depletion are in urgent demand. Cholesterol oxidase (3β-hydroxysteroid oxidase, EC 1.1.3.6) catalyzes the cholesterol oxidation to the intermediate 5-cholesterin-3-one and the O2 reduction to H2O2, and the steroid isomerization with a trans-A and B ring junction to reduce 4-cholesterin-3-one (Devi and Kanwar 2017).

Cholesterol oxidase acts as a bactericidal and plays a critical role in bacterial pathogenesis. In this study, from the concept that the probiotic produces immunomodulatory agents, such as levan, ten bacterial honey isolates were screened for levansucrase production and levan yield. Four isolates were selected based on high levansucrase production. Some probiotic characteristics were evaluated. Also, their ability to produce lipase and cholesterol oxidase were evaluated and their roles in lowering the cholesterol were evaluated.

## Materials and methods

### Bacterial isolation

#### Isolation of bacteria from different honey sources

Honey samples were collected at the mature stage from different sources; Nigerian mountain honey (isolate 9A) and Acacia (Australia) (isolates 10A), New El-Wadi (Dakhla) Egypt (2M, 13M). Serial dilutions were made for each honey sample (10–9). Then spread on nutrient agar (NG) or MRS plates and incubated at 37 °C for 24 h. One of the bacterial colonies was picked and purified by serial subculture and plating, then stored at − 80 °C in the NG or MRS medium containing glycerol (50%). Isolates were chosen for further morphological analysis and levansucrase activity.

### Levansucrase assay

Levansucrase assay was performed according to the method of Yanase et al. (1991). Culture filtrate, 0.5 mL, was incubated with 1 mL 20% sucrose and 0.5 mL 0.1 M acetate buffer at pH 5.2 and incubated at 30 °C for 15 min. The decreasing amounts of sugars produced were measured by glucose oxidase kits. One unit of enzyme activity was defined as the amount of enzyme that produced sugars equivalent to 1 μmol of glucose/min.

### Crude levan (CL) precipitation

The levan yielding organisms were cultivated on the levan producing medium by shaking flask cultivation technique. After the early stage of the stationary phase, the culture filtrate (CF) was centrifuged at 5000×*g* to get rid of bacterial cells. The CF was dialyzed against deionized water for 24 h by using a dialysis membrane (MWCO 14,000 Da, diameter 60 mm) to remove the unfermented sucrose, and any fermentation products had low molecular weights (MW). Levan was precipitated with three volumes of ice-cold ethanol (99%).

### Isolates identification

The isolated strains were examined by microscopic observation, and identification was based on morphological tests and 16S rRNA.

### Identification of bacterial isolates by 16S rRNA gene sequencing

The 16S rRNA gene fragments of the four levansucrase producing bacteria were amplified using the universal primers F-27 (5′-AGAGTTTGATCMTGGCTCAG-3′) and R1494 (5′-CTACGGYTACCTTGTTACGAC-3′) using PCR machine (Bio-rad T100 thermal cycler). The PCR products were checked via agarose gel electrophoresis then purified using a gel extraction kit and sequenced by Macrogenkoria.

### Phylogenic analysis of bacterial isolates

The evolutionary history was inferred using the Neighbor-Joining method. The tree was computed using the Maximum Composite Likelihood method. The analysis involved 8 nucleotide sequences of which four sequences of 16S rRNA gene amplified from bacterial isolates of current study while the other four sequences are representing the most similar hits were obtained from the NCBI gene bank database. Evolutionary analyses were conducted in MEGA5 software. The amplified region of the 16S rRNA segment from the isolates was submitted to NCBI Gen bank under Accession number: MT427639 of *B. subtilis* (9A), Accession number: MT427640, of *Achromobacter* sp. (10A), Accession number: MZ349055 of *Bacillus paranthracis* (13M), Accession number: MW325728 of *Bacillus paralicheniformis* (2M).

### Hemolytic activity

Each isolate was cultivated in Petri dishes containing nutrient agar (NG) for 9A and 10A or MRS for 2M and 13M supplied with 5% human blood and was grown at 37 °C for 24 h. The presence (or absence) of hemolysis was investigated visually (Chaiyawan et al. 2010).

### Antibiotic sensitivity

The pattern of resistance/sensitivity to the antibiotic of the isolated strains was tested using the disc diffusion method (Oxoid), as described previously. Four types of different antibiotic discs were used included Amoxicillin (Epico) + Flucloxacillin (Epico), Ampicillin (Epico), Gentamicin (Epico), Benzathine benzylpenicillin (Mup), Epicephin (Epico), Vancomycin (Mylan), Amikacin (Lldong pharma company), and Zino)l (Pharco p international. The producers are the tested isolates were activated for 24 h on NG or MRS according to the isolate type. A total of 100 μL of the diluted cultures (adjusting the optical density for each strain to 0.1 O. D) was diffused in nutrient agar or MRS. The different antibiotic discs were applied on the surface. The plates were incubated at 4 °C for 2 h. and were incubated at 37 °C and 24 h of inoculation. The zone diameter of inhibition (ZDI) values was measured.

### Catalase and protease tests

The catalase activity of the isolates was detected by resuspending the culture in a 3% solution of hydrogen peroxide. The protease activity was determined according to (Bendicho et al. 2002). The bacterial dilutions were streaked on casein agar plates for testing proteolytic activity, the plates were incubated for 24 h at 30 °C, and strains that produced clear zones in the medium were selected for further experiments.

### Resistance to low and high pH

The method of Conway et al. (1987) was used for the acid tolerance study of bacterial isolates. Where the freshly prepared cultures were transferred into the nutrient broth (NB) medium (5%) adjusted to pH 2.0 and pH 3.0 with 2 M HCl and pH 9 and pH11 with 1 M NaOH. They were then incubated at 37 °C and culture samples were taken after 1, 3, and 6 h. Medium neutralization was done by serial dilutions in phosphate buffer (0.1 M, pH 7.0) and re-culture on nutrient agar (NG) OR MRS plates. The plates were incubated at 37 °C for 24 h and survival (%) was determined by comparing the viable bacterial count after incubation at pH 2.0, 3.0, 9, and 11 compared to the control bacterial count incubated at pH 7.

### Bile salt resistance

Bile tolerance was conducted according to (Liong and Shah 2005; Westermann et al. 2016) where the four isolates were grown overnight at 37 °C in MRS (2M,13M) or nutrient broth medium (9A, 10A) supplemented with 0.3% (w/v) bile salt (Oxgall, USA). Then samples were incubated at 37 °C for 3 h, 6 h, 24 h, and slants without the bile salt were left as a control. A spectrophotometer (O.D. at 660 nm) was used to detect bacterial growth. The ratios of bile salt resistance were calculated as follows: Percentage of surviving cells incubated with bile to the cell count of control.

### Hydrophobicity and auto-aggregation

The medium used in both tests is nutrient agar (NG) for 9A and 10A or MRS for 2M and 13M at 37 °C for 24 h. Cell Hydrophobicity Assay in vitro method (Rosenberg et al. 1980) was used to assess the bacterial adhesion to hydrocarbons (Tween 80). The fraction of adherent cells was calculated as a percent decrease in absorbance of the aqueous phase as compared to that of original cell suspension. Cell surface hydrophobicity or the percent adhesion was calculated by the following formula:

$$\% {\text{ Adhesion }} = {\text{ initial OD}} - {\text{ final OD }} \times { 1}00{\text{ initial OD}}$$

The auto-aggregation method was done according to Zuo et al. (2016). The cells were washed twice using phosphate buffer (pH 6.6) and adjusted to the optical density of 0.60 at 600 nm (A0, H0). For the auto-aggregation assay, each bacterial suspension (8 mL) was incubated at 37 ◦C and the auto-aggregation values were measured at 24 h (Ht).

$${\text{H }} = \, \left( {{\text{H}}0 \, - {\text{ Ht}}} \right)/{\text{H}}0 \, \times { 1}00\%$$

### Antimicrobial activity

Antimicrobial activity of the isolates was carried out by agar well diffusion method (Mishra and Prasad 2005) using cell-free culture supernatants (CFCS) of the isolated probiotic strains against pathogenic indicator bacteria: *Staphylococcus aureus*, *Bacillus cereus*, *Candida albicans* NRRL Y-477, *Aspergillus niger* NRRL 599 and *Escherichia coli* MC1400. Wells of 5 mm diameter were prepared and loaded with a volume with 100 μL of CFCS of each honey isolate and marked adequately with the isolates’ names. The plates were kept for 2 h at room temperature, then incubated for 24 h at 37 °C. The zone diameter of inhibition (ZDI) values was measured. The tests were performed in triplicate and the data were represented with mean ± SD.

### Antioxidant activities and H_2_O_2_ tolerance

#### 1.1‐Diphenyl‐2‐picryl‐hydrazine (DPPH) assay

The free radical scavenging activity using the DPPH reagent was determined as described previously [21]. The cells free supernatant of each isolate (100 µg) was added to 1.0 mL of freshly prepared methanolic DPPH solution (20 µg/mL) and stirred. The decolorizing processes were recorded after 5 min of reaction at 517 nm and compared with a blank control.

Antioxidant activity = [(control absorbance − sample absorbance) /control absorbance] × 100%

The tolerance of strains to H2O2 was assessed by the method of Li et al. (2012) but with only 30 min incubation time. Overnight grown cultures of the isolates were inoculated (1% v/v) into NG medium or MRS (control) and the two mediums containing 0.1% hydrogen peroxide and incubated at 37 °C for 30 min.

### Cholesterol determination

Cholesterol removal by tested bacterial isolates was determined according to the manufacture’s kits (Bio-diagnostic company for the diagnostic and research reagents). The cholesterol is determined after enzymatic hydrolysis and oxidation. The quinone imine is formed from hydrogen peroxide and 4- amino antipyrine in the presence of phenol and peroxidase. For HDL determination phosphotungstic acid and magnesium ions selectively precipitating all lipoproteins except the HDL fraction—cholesterol present in the supernatant can be determined by the same method used for total cholesterol. Low-density lipoproteins (LDL) are precipitated by heparin at their isoelectric point (pH 5.04). After centrifugation, the high-density lipoproteins (HDL) and the very low-density lipoproteins (VLDL) remain in the supernatant. These can then be determined by enzymatic methods.

$${\text{LDL - cholesterol}} = {\text{ Total cholesterol }}{-}{\text{ cholesterol in the supernatant}}.$$

### Cholesterol oxidase assay

The activity of the extracellular CO enzyme was determined according to the method described by Inouye et al. (1982). Briefly, 0.1 mL of culture supernatant was added to 0.4 mL of 125 mM Tris–HCl buffer (pH 7.5). The mixture was incubated in a water bath at 37 °C. After 3 min, 25 µl of 12 mM of cholesterol in isopropanol solution was added to the mixture, and incubation proceeded for a further 30 min. Afterward, 2.5 mL of absolute ethanol was added to the reaction medium, and then the amount of formed 4-cholesten-3-one was determined spectrophotometrically by measuring the absorbance at 240 nm. Reaction blanks were prepared by cholesterol solution with isopropanol. One unit of cholesterol oxidase activity (U) was defined as the amount resulting in the formation of 1 µmol of 4-cholesten-3-one in 30 min at 37 °C (concentration of 4-colesten-3-one was calculated from a standard curve previously prepared with serial dilutions (10–100 µg) of 4-cholesten-3-one dissolved in isopropanol.

### Lipase assay

One mL of the culture filtrate of each bacterial isolate was incubated separately at 37 °C with a mixture containing 1 mL of 0.1 M Tris–HCl buffer (pH 8.0), 2.5 mL deionized water, and 3 mL of vegetable oil. After 30 min, each test solution was transferred to a 50 ml Erlenmeyer flask and 3 mL of 95% ethanol was added to terminate the reaction. The formed fatty acids were titrated using 0.1 M NaOH and phenolphthalein as an indicator turning pink at the endpoint. Both test and blank were performed. Enzyme activity was expressed as units per mL enzyme (Sayali et al. 2013). One lipase unit is defined as the quantity of enzyme that will liberate 1 μmol of butyric acid per minute under the conditions of the test.

## Results

### Screening for levansucrase productivity

Ten isolates as two groups of bacteria were isolated from different honey sources using both nutrient agar medium (A) and MRS agar medium (M).

**Fig. 1:**
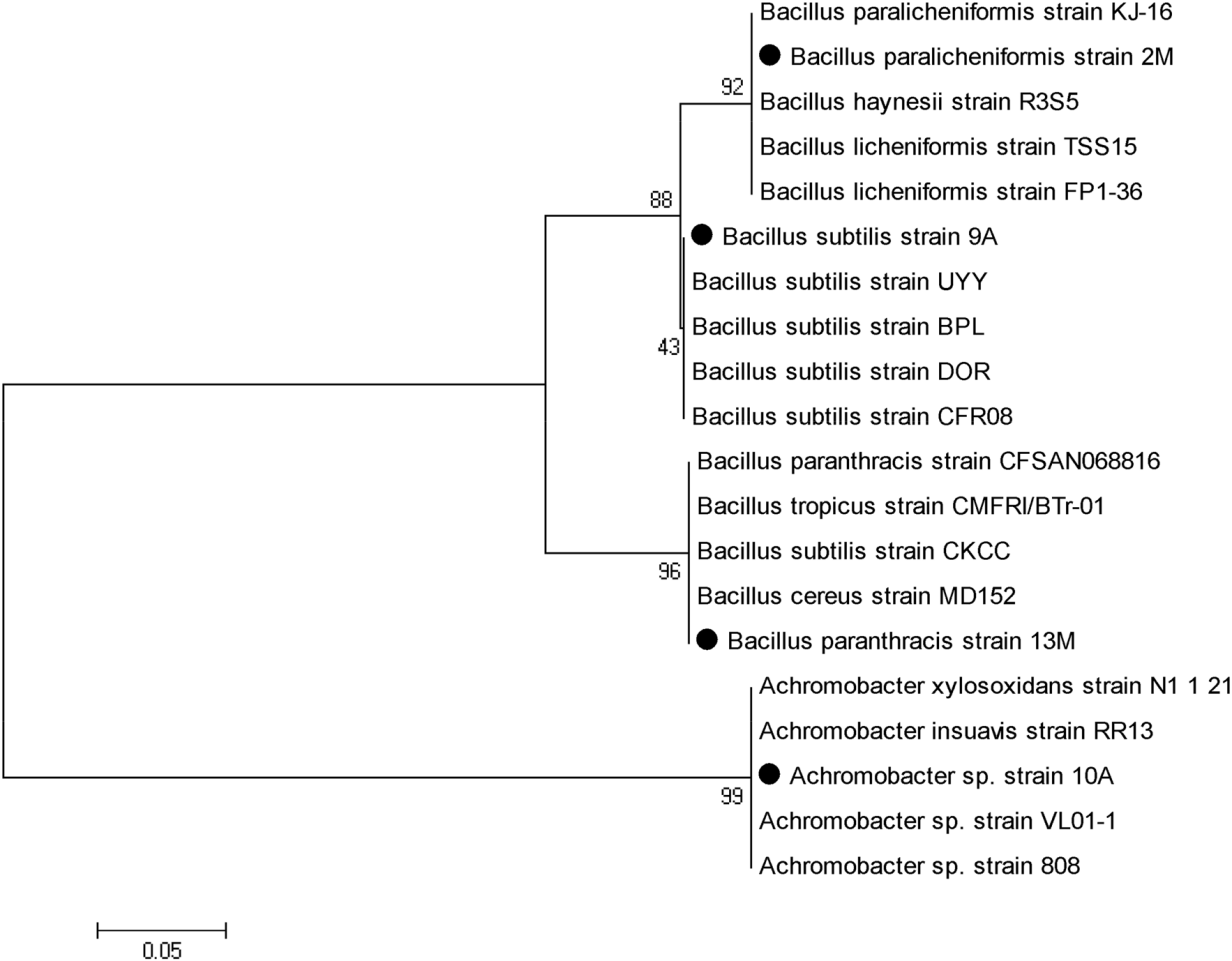
Neighbor-joining phylogenetic tree based on 16S rRNA gene sequences of four levansucrase-producing bacterial isolates obtained from different honey samples. Dark circles represent isolates obtained in this study

**Fig. 2:**
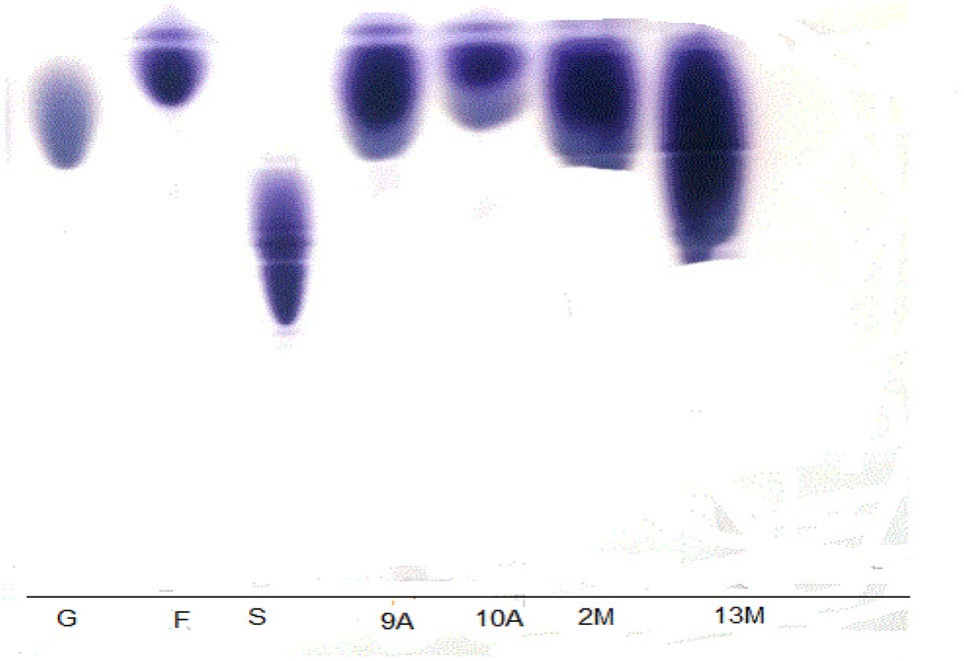
TLC for identification of polymer yielded by the levansucrase producing isolates after acid hydrolysis of the precipitate by HCl

The results in Table 1 showed that all the isolates were levansucrase producers with a wide degree of variations. The highest levansucrase activity was noticed in isolates 10A and 9A (31.85 U/mL and 20.30 U/mL, respectively) which were cultivated in nutrient agar, and isolates 2M, 13M (18.30, 6.00 U/mL, respectively) which cultivated on MRS agar medium. There is no clear relation between the final pH and enzyme production. But generally, the optimum pH value of levansucrase production is pH 7. The most potent four isolates (9A, 10A, 2M, and 13M) were identified based on 16 s rRNA (Fig. 1) as *Achromobacter* sp. (10A), *Bacillus paralicheniformis* (2M), *Bacillus subtilis* (9A), *Bacillus paranthracis* (13M) and chosen for further investigation. The produced levans for the four isolates were precipitated from the culture filtrates and their hydrolysis products were identified by thin-layer chromatography (TLC). The results in Fig. 2 indicated that fructose was the main backbone in all the isolates.

**Table 1:** Survey on 10 bacterial cultures to produce levansucrase using shaken culture technique at 37 °C

Bacterial isolates	Levansucrase activity (U/mL)	Protein (U/mg protein)	Specific activity (U/mg)	Final pH
2M	18.30 ± 0.15	0.38	48.15	6.21
9M	0.015 ± 0.09	0.11	0.13	7.80
8M	0.15 ± 0.12	1.25	0.12	4.24
1M	0.03 ± 0.04	0.29	0.10	7.19
13M	6.00 ± 0.06	1.1	5.45	7.26
9A	20.30 ± 0.17	0.72	28.20	7.40
5A	18.67 ± 0.05	0.80	23.34	6.70
13A	6.24 ± 0.11	1.14	5.47	4.70
8A	19.59 ± 0.18	0.81	24.19	7.40
10A	31.85 ± 0.20	1.18	27.00	6.98

### Determination of levan amounts

The levan separated from the culture filtrate was dried in Petri dishes, collected and their dry weight was determined. The highest amounts of the crude levan were obtained with the isolates 2M (48 g/L), and the lowest weight was detected with the isolate 10A (24.44 g/L). While isolates 9A and 13M recorded 46 and 44.4 g/L, respectively.

### Hemolysis property

The results showed that all the tested isolates did not affect blood and no Hemolysis activity was detected in all the used isolates.

### Antibiotic resistance

**Fig. 3:**
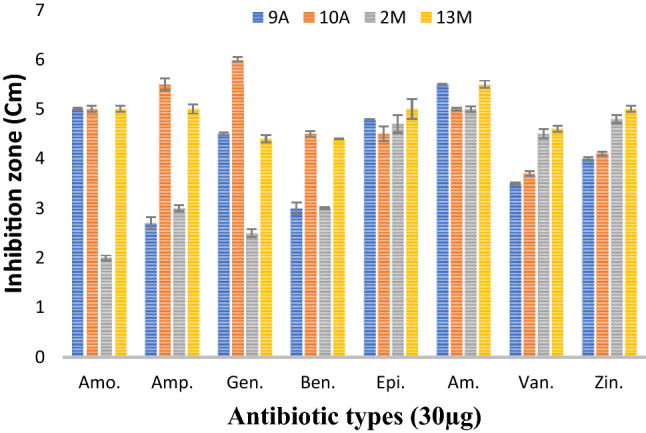
Effect of different antibiotics on isolates surviving

The results in Fig. 3 showed that all the tested bacteria were sensitive against Amoxicillin + Flucloxacillin, Ampicillin, Gentamicin, Benzathine benzylpenicillin, Epicephin, Vancomycin, Amikacin, and Zinol recorded promising inhibition zone. The inhibition zone for the isolate 13M ranged from (4.4–5.0 cm). While isolate 2M showed the lowest inhibition zone with all the tested antibiotics (2.0–3.0 cm).

### Catalase and protease tests

In Table 2. The result showed that the highest catalase activity was obtained by 9A and 10A while 13M and 2M had a weak activity. On the other hand, isolate 2M recorded strong protease activity. While 9A and 10A had a moderate activity and 13A appeared weak activity.

**Table 2:** Catalase and protease activity for the honey isolates

Isolates NO	9A	10A	13 M	2 M
Enzymes				
Catalase	+ 3	+ 3	+	+
Protease	+ 2	+ 2	+	+ 3

+  = weak

+ 2 = moderate

+ 3 = strong

### Acid and base tolerance

In this experiment, the isolates were incubated in acid and alkaline conditions at different time intervals. The results in Table 3 showed that isolates had a resistance to the severe acidic and basic conditions with the degree of difference. Isolate 13M could keep 72% of its initial viability at pH 2 after 6 h., while it kept most of its viability at pH 7. Also, 9A and 10A maintained their viability after 6 h of incubation at pH 9 and pH 11, while they lost about 42% and 68% of their viability at pH 2 and pH 3 after 6 h., respectively. Isolate 2M kept 57.4% of its viability at pH 2 after 6 h. In the basic case, it lost only 6- 10% of its viability at pH 9 and pH 11.

**Table 3:** Isolates tolerance to acidic pH (pHs 2 and 3.5) and alkaline pH (pHs 9 and 11) for 3 and 6 h and the bile salt for 24 h

Bacterial isolates	Survival (%)												
	pH 11			pH 9			pH 3			pH 2			Bile salt 0.3%
	1 h	3 h	6 h	1 h	3 h	6 h	1 h	3 h	6 h	1 h	3 h	6 h	24 h
Control	100	100	100	100	100	100	100	100	100	100	100	100	100
2M	100	100	99.57	100	99.00	94.53	87.39	58.26	57.00	59.13	58.26	57.39	100
13M	100	98.36	91.00	94.26	93.44	90.57	75.13	74.05	73.51	78.00	73.51	72.43	100
9A	100	100	100	100	100	100	100	100	88	98.09	96.56	58.01	100
10A	100	100	100	100	100	100	37.30	31.11	29.00	38.54	37.00	32.00	100

### Bile salt resistance

The isolates with 0.03% bile salt for 3, 6, and 24 h. The result in Table 3 showed that all isolates showed complete tolerance to the bile salt until 24 h.

### Antimicrobial activity

The results in Table 4 showed that all the cell-free supernatant of the isolates recorded antimicrobial activities against *C. albicans* and *B. subtilis* with various degrees. Only 9A and 13M recorded inhibition zone against *A. niger* of (3.3 and 5.0 cm), respectively. Also, 2M and 13M caused strong inhibition to the *S. aureus* of (4.10 and 4.0 cm), respectively. Also, *E. coli* was inhibited by 10A (3.1 cm) and 2M (3 cm).

**Table 4:** Antimicrobial activities of the four probiotic isolates

Bacterial isolates	*Bacillus cereus*	*Candida albicans* NRRL Y-477	*E. coli* MC1400	*Staphylococcus aureus*	A. *Niger* NRRL 599
	Antimicrobial activity (cm)				
9A	2.60 ± 0.02	3.00 ± 0.05	No inhibition	No inhibition	3.30 ± 0.11
10A	2.80 ± 0.04	2.80 ± 0.07	3.10 ± 0.08	No inhibition	No inhibition
2M	3.50 ± 0.15	2.00 ± 0.09	3.00 ± 0.09	4.10 ± 0.13	No inhibition
13M	3.30 ± 0.25	3.50 ± 0.12	No inhibition	4.00 ± 0.18	5.00 ± 0.07

### Hydrophobicity and auto-aggregation

**Fig. 4:**
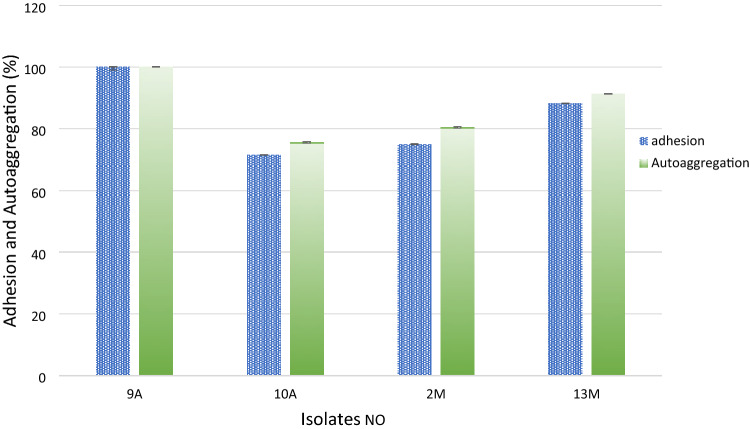
Evaluation of the isolates hydrophobicity and auto aggregation capacity

The result Fig. 4 exhibited that all the isolates had great hydrophobicity and Auto-aggregation. The isolate 9A showed complete hydrophobicity and Auto-aggregation. In general, all the other isolates recorded more than 70% hydrophobicity and Auto-aggregation.

### H_2_O_2_ tolerance

**Fig. 5:**
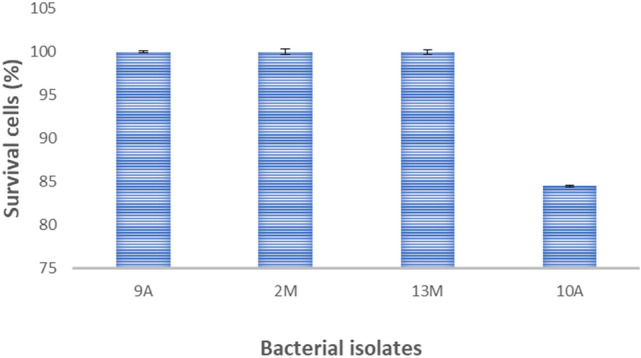
Tolerance of the isolates in medium supplemented with 0.1% H_2_O_2_ for 24 h

All the isolates showed complete withstood 0.1% H_2_O_2_ except 10A recorded 85% surviving (Fig. 5).

### Antioxidant activity

**Fig. 6:**
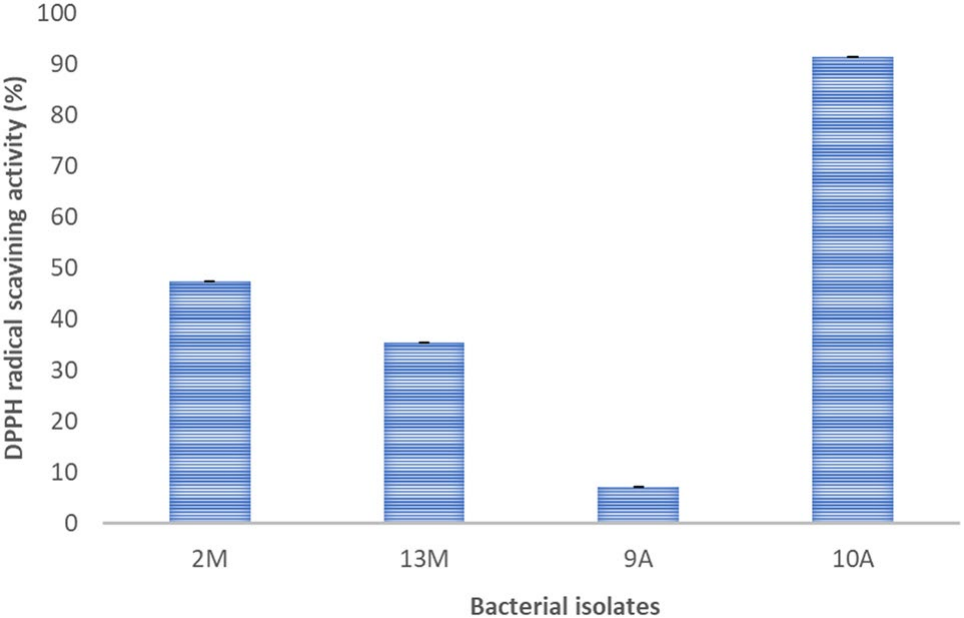
DPPH free radical scavenging activity for the cell-free supernatants of the four isolates

The antioxidant activity showed that all the isolates had an antioxidant activity with varying degrees. Isolate 10A showed the highest reducing power activity 91.45% while isolate 9A recorded only 7.24% activity (Fig. 6).

### Reduction of total cholesterol by the isolated probiotic bacteria

**Fig. 7:**
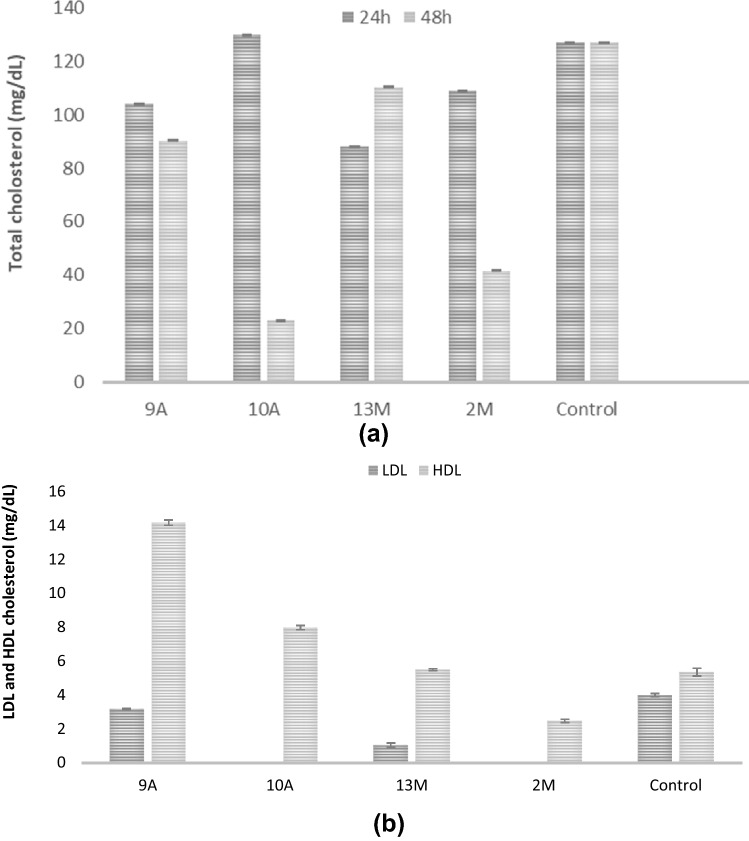
Determination of the total cholesterol (mg/dL) (**a**), The isolates influence LDL and HDL amounts after 24 h (**b**)

The results (Fig. 7a) showed that all the isolates reduced the total cholesterol quantity to great instant. Isolates 10A and 2M led to 75%, 60% reduction in cholesterol, respectively after 48 h. While isolate 13M reduced the cholesterol content from 127 (mg/dL) to 23.9 (mg/dL), while isolate 9A showed a slight reduction after 24, 48 h (Fig. 7a).

### Effect of the four probiotic isolates on LDL and HDL cholesterol (mg/dL) amount

All the isolates showed diminish in LDL amount with varying degrees. Isolates 10A and 2M reduced LDL completely, while 9A slightly lowered the LDL level (25%) (Fig. 7b). Also, the result showed a significant increase in HDL levels in the case of isolates 9A and 10A. On contrary, isolate 2M showed a reduction in HDL amounts to 46%. Isolate 13M showed a non-noticeable increase.

### Lipase activity of the four probiotic isolates

All the isolates were screened for lipase production at 24 and 48 h. The results in Table 5 showed that isolate 9A showed the highest lipase activity (84 U/mL) while the lowest activity was produced by isolate 13M (9 U/mL) after 48 h.

**Table 5:** Evaluation the isolates’ ability to produce cholesterol oxidase and lipase

Bacterial isolates	Cholesterol oxidase (µg/mL)		Lipase (U/mL)	
	24 h	48 h	24 h	48 h
9A	19.75 ± 0.02	115.39 ± 0.05	1.2 ± 0.07	84 ± 0.08
10A	41.53 ± 0.12	132.46 ± 0.11	1.2 ± 0.09	20.4 ± 0.06
13M	15.99 ± 0.15	158.80 ± 0.19	1.8 ± 0.15	9.0 ± 0.09
2M	0.0	105.31 ± 0.25	2.4 ± 0.14	10.2 ± 0.10

### Cholesterol oxidase activity of the four probiotic isolates

Also, the isolates were screened for cholesterol oxidase activities. They recorded 115.39, 132.46, 158.80 and 105.31 (µg/mL) for 9A, 13A, 13M and 2M, respectively (Table 5).

## Discussion

Probiotics are those bacteria that live in the intestine and provide great benefit to it. The importance of beneficial bacteria is not less than the importance of the body's organs. Therefore, their balance must be maintained in the living body, as an imbalance in them may result in many dangerous diseases, such as the immune system and cancer diseases. Polysaccharides (PS) production is the famous criterion in some types of probiotic bacteria (Angelin and Kavitha 2020; Stack et al. 2010). Some PS act as prebiotics and play a role in increasing the probiotic bacteria count and keeping their balance. Accordingly, ten bacterial strains were isolated from various honey sources. Previously, honey was identified as a new reservoir for the probiotic strains (Esawy et al. 2012; Hamdy et al. 2017, 2018). Among the ten isolates, four isolates recorded maximum levansucrase production with high levan yield. Hamdy et al. (2017, 2018) found that the best bacterial strains that gave the characteristics of beneficial bacteria were the levansucrase producers and accordingly, the selection of a probiotic bacteria was done based on levansucrase production. Previously, most of the bacteria isolated from honey form the levan polymer by the action of a levansucrase (Salama et al. 2019; Esawy et al. 2011, 2016; Gamal et al. 2020). Levan known as a prebiotic could increase the beneficial bacteria in the stomach and has a beneficial effect on public health protection (Ragab et al. 2020). The four isolates were identified as *Achromobacter* sp. (10A), *B. paralicheniformis* (2M),* B. subtilis *(9A),* B. paranthracis* (13M). The European Food Safety Authority (EFSA) confirmed that the absence of hemolytic activity is an urgent demand for probiotic selection and use in food products. In this work, the hemolytic activity was negative in all the isolates. This result is coincided with (Oh and Jung 2015; Yasmin et al. 2020). Such strains are non-virulence and lack hemolysin confirmed that the virulence does not appear among the bacterial strains (FAO/WHO 2006). Another important parameter for the evaluation of probiotic safety is antibiotic sensitivity. Probiotic bacteria which have sensitivity to antibiotics, could proliferate in the gut, and maintain microbial balance, thereby reducing opportunistic microorganisms (Shobharani and Agrawal 2011). But the risk lies in the transfer of their plasmids with a resistant gene to the pathogenic bacteria. This may cause severe problems (Cristino 1999). The results referred to the high sensitivity of the four isolates to the tested antibiotics. This result recommended the four isolates be used safely in pharmacochemical and food sections. The tested isolates showed significant catalase activity. Catalase could reduce the bad effects of active oxygen molecules or free radicals generated during the metabolic processes. Free oxygen radicals are injurious to the host's health. Since the potent probiotic strain inhabiting the gut could act as a good antioxidant (Patel et al. 2009). All isolates were examined for alkaline protease production, in plate assay. Proteases accelerate the protein digestion process. Also, they played a significant role as defense mechanisms against pathogens by cleaving their receptor sites in intestinal epithelial cells (Patel et al. 2009). The four isolates had a high tolerance to the H_2_O_2_, this could be back to the presence of catalase or peroxidase enzyme (Esawy et al. 2012). The antimicrobial activity is considered one of the most important features for probiotic assessment. It permits them to make a balance between the beneficial and the pathogenic bacteria. In this result, all the examined isolates had antimicrobial activity against *C. albicans*. A similar result was obtained by Hamdy et al. (2017) where among 6 bacterial honey isolates, four isolates recorded antimicrobial activities against *C. albicans* NRRLY-477. This result paid attention to the honey isolates as an efficient antimicrobial agent against the *C. Albicans* is an opportunistic yeast pathogen that could colonize the human tissue and cause serious human disease (Hernday and Johnson 2010). In this study 10A and 2M play promising roles in *E.* *Coli* inhibition. Five of the *Lactobacillus* strains inhibited carbapenem-resistant *E. coli* (CRE316) and *Klebsiella pneumonia* (CRE632) growth (Chen et al. 2019). There is a multitude of probiotic formulations that are supposed to benefit human health, including immunostimulatory effects or interbacterial competition between beneficial and pathogenic bacteria (Piewngam et al. 2018). Isolates 13M and 9A have significant activities against the *A. niger* and the other two isolates lack the activity completely. The spoilage and toxicity of fungi like *A. niger* happen during food storage and maintenance of food products. Moreover, *A. niger* produces allergen spores and mycotoxins that seriously threaten human health (Twarużek et al. 2020). The cell-free supernatant of the isolates 2M and 13M showed strong activities against *S. aureus*. Piewngam et al. (2018) pointed to a firm relationship between the presence of Bacillus bacteria and the absence of *S. aureus*. Until this moment no effective vaccine against *S. aureus* has been approved. Also, all the isolates showed antagonistic activity against *B. subtilis* with varying degrees. The isolate cells' hydrophobicity was studied using hexadecane. Our results refer to the high hydrophobicity ability for all the tested isolates. Hydrophobicity is an important parameter that could evaluate bacterial adhesion and colonization in the gastrointestinal tract (Zavaglia, et al. 2002). The acid and base environment is the most factors that reduce the bacterial population in the gastrointestinal tract (GIT). Survival in acid may have clinical significance because the probiotic bacteria should pass through the stomach at pH less than 3 for up to 2 h. before colonizing the intestinal tract. Within this context, the four bacteria were subjected to pH 2, 3, 9, and pH 11. The tested isolates showed high tolerance to both the basic and acid mediums. It was noticed that spores of *Bacillus *sp. isolate NO JHT3, DET6, and DET9 were found to be good tolerant to both partially simulated acid conditions (Patel et al. 2009). High alkali tolerance was exhibited by strains of *Lactobacillus* spp. that originated from plant material, with pH values between 8.5 and 8.9 (Sawatari and Yokota 2007). The four isolates exhibited complete tolerance to the bile salt. These results confirm the suggestion that honey carries spores with unique properties. Also, the results support an effective probiotic formulation system with a high number of viable cells, and its protective effect can be leveraged in the development of probiotic products with health benefits. The isolates had antioxidant activities with different levels. Oxidative stress is considered a risk factor in the pathogenesis of numerous chronic diseases, such as asthma, inflammatory arthropathies, diabetes (Chiavaroli et al. 2011; Kanwar et al. 2009). Previously, six *Bacillus* sp. isolated from different honey sources were recorded as strong antioxidant agents (Esawy et al. 2012; Hamdy et al. 2018)

The results recorded cholesterol oxidase activities in all the isolates with different degrees. The presence of this enzyme could explain the high antimicrobial activity in the tested isolates. The four isolates were lipase producers, and the maximum activity was obtained with isolate 2M after 24 h and isolate 9A after 48 h. The results showed the ability of the four isolates to reduce the total cholesterol and the LDL amounts, while there is an increase in HDL amount to some extent except the isolate 2M. Cho and Kim (2015) suggested the probiotic bacteria could play a role in lipid metabolism by lowering total cholesterol and LDL concentration. Nine probiotic organisms of the genera Lactobacillus and Bifidobacterium contain digestive enzymes revealed significant decreases in blood concentrations of low-density lipoprotein (LDL) and discussed the role of lipase as a central enzyme in lipid metabolism (Ichim et al. 2016). Previously, lipase produced from the probiotic bacteria *Lactobacillus Plantarum* and *Lactobacillus Brevis* was used for biodiesel formation (Khan et al. 2020). Previously, it was reported in low molecular weight *B. subtilis* levan SL1 that showed high selective cytotoxicity against HepG2 cells (Abdel-Fattah et al. 2012).

## Conclusion

Amidst the rising need for non-dairy probiotic foods. This study focused on the isolation of probiotic bacteria from different honey sources and the four bacteria which had the highest levansucrase activity were identified and selected for the probiotic study. This study pointed to the four isolates as safe strains distinguished by promising probiotic characters. The results referred to the role of bacteria in lipid profile and in lowering the harmful cholesterol and recommended these types of bacteria to be used in different food and pharmaceutical aspects.
